# Decoding unintentional weight loss: How the right questions make a difference

**DOI:** 10.1016/j.clnu.2025.04.009

**Published:** 2025-04-10

**Authors:** Jos W. Borkent, Barbara S. van der Meij, Marian A.E. de van der Schueren

**Affiliations:** a Department of Nutrition, Dietetics and Lifestyle, School of Allied Health, HAN University of Applied Sciences, Nijmegen, the Netherlands; b Department of Human Nutrition and Health, Wageningen University and Research, Wageningen, the Netherlands

**Keywords:** GLIM, Involuntary weight loss, Screening tools, Misclassification, Body image

## Abstract

**Background/aims:**

Malnutrition screening tools often include questions about unintentional weight loss (UWL). It is unclear whether individuals who intentionally desire to lose weight or perceive themselves as overweight interpret questions regarding UWL accurately. We assessed potential misclassification of UWL-related questions in these groups.

**Method:**

Data from the Lifelines cohort was used (ñ125.000, age >18 years). Prevalence ratios (PRs) were calculated with a simple question regarding UWL as the dependent variable and the desire to lose weight and perception of one’s body weight (too heavy vs. just right/too light) as separate independent variables. Associations were stratified by body mass index (BMI) group (normal weight vs. overweight/ obesity) and adjusted for various comorbidities, demographics, and quality of life.

**Results:**

Of participants with normal weight, 30.3 % desired to lose weight, 37.9 % perceived their body weight as too heavy and 5.1 % reported UWL; this was 80.1 %, 92.7 % and 2.7 % for those with overweight/ obesity. In both BMI groups, the prevalence of UWL was ∼60 % lower in participants who desired to lose weight or perceived themselves as too heavy (prevalence ratios 0.40−0.43, p-value <0.05).

**Conclusion:**

Since there is no logical basis for the desire to lose weight or one’s perception of body weight to prevent UWL, lower reported prevalence rates on UWL-related questions are likely the result of misinterpretation. Malnutrition screening tools that include questions regarding UWL might underestimate the prevalence rates of malnutrition in higher BMIs, as individuals in these groups often aspire to lose weight or perceive themselves as too heavy.

## 1 Introduction

In Western society, malnutrition is a relatively common problem, often associated with disease and/or older age [1]. With increasing life expectancy and more older individuals living with multiple chronic diseases, it is expected that this problem will escalate in the coming years. At the same time, the population is also becoming heavier, with obesity rates expected to affect more than 50 % of the Western population [2]. Although this may seem contradictory, malnutrition and obesity can occur simultaneously within the same individual [3].

Malnutrition screening tools are important to identify individuals at malnutrition risk. The ideal screening tool includes simple, easily measurable questions that can be assessed collaboratively by healthcare providers and patients. Multiple studies have shown that unintentional weight loss is a marker of malnutrition and is associated with poor clinical outcomes. Therefore, almost every malnutrition screening tool includes a question about unintentional weight loss [4].

From the perspective of healthcare professionals, questions regarding unintentional weight loss may seem relatively straight-forward. However, in a recent study aimed at developing a Malnutrition Awareness Scale (MAS) for older adults, participants often struggled to understand the term ‘unintentional weight loss’. The final version of the MAS therefore includes an explanation with an introductory sentence to avoid confusion *(*‘*Unintentional weight loss*’ *refers to weight loss without any conscious effort on your part. This questionnaire is about this type of weight loss. This questionnaire is not about* ‘*intentional weight loss’, in other words, weight loss because you have been dieting or exercising more)* [5].

The fact that weight loss can be interpreted in multiple ways is probably even more confusing for individuals who desire to lose weight or those who perceive themselves as too heavy. They typically find weight loss pleasing, especially if it happens relatively easily, as they may have been actively trying to lose weight for years. So how unintentional is unintentional weight loss for them? When the desire to lose weight is strong, individuals might perceive any weight loss as a positive outcome, even if it is unintended from a medical point of view. Thus, we hypothesize that the question on unintentional weight loss, which is incorporated in almost every malnutrition screening tool, will result in an underestimation of the actual number of individuals experiencing unintentional weight loss in individuals who perceive themselves as too heavy or desire to lose weight.

In this study, we assessed if prevalence rates of unintentional weight loss could be misclassified by the desire to lose weight or perception of body weight in individuals with normal weight and those with overweight or obesity.

## 2 Methods

Data was used from lifelines: a multi-disciplinary prospective population-based cohort study examining in a unique three-generation design the health and health-related behaviors of 167,729 persons living in the North of the Netherlands. It employs a broad range of investigative procedures in assessing the biomedical, socio-demographic, behavioral, physical and psychological factors which contribute to the health and disease of the general population, with a special focus on multi-morbidity and complex genetics.

Recruitment of participants took place between 2006 and 2013 and was carried out by general practitioners (GPs), through included family members and self-registration. There were no specific inclusion criteria, but individuals were excluded if they had terminal illnesses (life expectancy <5 years), severe mental illness (i.e., not fully capable of making rational decisions), were not able to fill out the questionnaire, or not able to understand the Dutch language [6].

For this specific study, only the first assessment was used, which included questions on unintentional weight loss, desire to lose weight and perception of body weight.

### 2.1 Inclusion criteria

Adults (>18 years), not following a diet, availability of data on: BMI, unintentional weight loss, perception of body weight and desire to lose weight.

### 2.2 Measurements

Anthropometric measurements were performed by a trained research nurse in a research facility. Height without shoes was measured with a SECA222 stadiometer and weight without shoes and heavy clothing with a SECA 761 scale. BMI was calculated from weight and height and categorized into normal weight 18.5−25 kg/m^2^ for those aged <65 years, 20−25 kg/m^2^ for those aged 65−70 years and 22−27 kg/m^2^ for those aged >70 years [7]. Participants with a BMI above these cut-off points were classified as having overweight/obesity.

After the physical examination, questionnaires were provided to participants to be filled in at home. These questionnaires included, amongst others, general demographic information, health and disease status, lifestyle and psychosocial factors.

For our analyses on unintentional weight loss in relation to the desire to lose weight or perception of one’s weight, we used the following three questions:

For unintentional weight loss: “*Have you lost a lot of weight recently without wanting to (*6 kg *in* 6 months *or* 3 kg *in one month)?*” (yes/no).

For the desire to lose weight: “*Do you want to lose weight*” (yes/no).

For perception of body weight: “*What do you think of your body weight?*”. This item was scored on a 5-point scale. We dichotomized the categories as much too heavy, too heavy, a bit too heavy into ‘too heavy’ and combined the ‘just right/too light’ into one category.

### 2.3 Statistical methods

Descriptive statistics were used to provide an overview of the included participants. Results were stratified by BMI group (normal weight vs. overweight/obesity). For the prevalence of unintentional weight loss, we used cross tabs, stratified by ‘desire to lose weight’ and ‘perception of own body weight’.

Poisson regression with a robust estimator for standard errors was used to assess the association between the independent variables (desire to lose weight and perception of body weight) and the dependent variable (unintentional weight loss). Poisson regression with robust standard estimators provides prevalence ratios (PRs) which are more representative than odds ratios (ORs) [8]. PRs (and corresponding confidence intervals) can be interpreted similarly to ORs: PRs between 0 and 1 indicate lower prevalences (with 1 − PR providing the relative percentage difference between groups), while PRs above 1 indicate higher prevalences (with PR − 1 providing the relative percentage difference between groups).

To assess potential misclassification (the incorrect labelling of participants as ‘not experiencing unintentional weight loss’), those with ‘no desire to lose weight’ or those who perceived their body weight as ‘just right’ were used as reference categories.

To adjust for possible confounding, analyses were adjusted for six common self-reported diseases (arthritis, burn-out, COPD, diabetes, cancer and heart attack), demographics (age, gender, employment status), Lifelines diet score (based on a 110-item FFQ, groups were stratified in quintiles) [9], stress level (single question on 10-point scale) and Quality of life scores (RAND-SF Health survey) [10] for physical function, health perception, vitality status, mental health, pain and social interactions. To assess PRs if the association differed between BMI groups, effect-modification terms were made (desire to lose weight * BMI category and perception of own body weight * BMI category). Sensitivity analyses were performed to assess whether effect sizes differed between those with overweight/obesity. A p-value of <0.05 was considered statistically significant.

## 3 Results

In total, ∼125.000 participants were included in the study, with a mean age of 44.8 years (SD: 13.0). More than half of them were female (58.8 %), and just over half of the participants were categorized as having overweight/obesity (54.0 %). Additionally, 3.7 % of participants reported unintentional weight loss.

### 3.1 Unintentional weight loss and the desire to lose weight

Approximately 30 % of participants from the normal BMI group desired to lose weight, vs. ∼80 % in the overweight/obesity group. Unintentional weight loss was reported by 5.1 % of participants in the normal BMI group and 2.7 % in the overweight/obesity group (p-value for between-group difference <0.001) (See Table 1).

In both BMI groups, the reported prevalence of unintentional weight loss was approximately 60 % lower in participants who desired to lose weight compared to those who did not desire to lose weight; normal BMI 2.4 % vs 6.0 % (adjusted PR: 0.40 (95 %CI: 0.36; 0.45) and overweight/obesity 2.2 % vs 4.5 % (adjusted PR: 0.42 (95% CI: 0.38; 0.42)). As anticipated, given the relatively comparable effect sizes between the normal-weight and overweight/obesity groups, no effect modification was observed between the two groups (P = 0.871).

Sensitivity analyses were performed to assess if the effect size for the association between the desire to lose weight and unintentional weight loss differed between those with overweight vs. obesity. Effect sizes were relatively comparable with PR: 0.40 (95% CI: 0.35; 0.45) for overweight and PR: 0.42 (95%CI: 0.32; 0.55) for obesity. Thus, no effect modification was seen between both groups (p = 0.60).

### 3.2 Unintentional weight loss and perception of body weight

Most individuals perceived their weight to be too high; 37.9 % in the normal weight group and 92.7 % in the overweight/obesity group (p < 0.01) (see Table 2).

Again, the reported prevalence of unintentional weight loss was approximately 60 % lower in participants who perceived themselves as too heavy, compared to those who perceived themselves as just right/too light; for normal BMI 2.6 % vs 6.4 % (adjusted PR: 0.40 (95 %CI: 0.37; 0.44) and overweight/obesity 2.4 % vs 5.4 % (adjusted PR: 0.43 (95 %CI: 0.37; 0.49)). No effect modification was seen between those with normal BMI vs. those with overweight/obesity (P = 0.934).

Sensitivity analyses were performed to assess if the effect size for the association between perception of body weight and unintentional weight loss differed between those with overweight vs. obesity. Effect sizes were relatively comparable in the overweight and obesity population with respectively 0.39 (0.34; 0.46) and 0.35 (95 %CI: 0.15; 0.82). No effect modification was seen between overweight and obesity (p = 0.38).

Given the strong correlation between the desire to lose weight and perception of body weight (Phi coefficient: 0.77), we evaluated which factor was the strongest predictor of unintentional weight loss by including both variables in a single model.

The PRs, adjusted for confounders, attenuated, but the association remained significant. In the normal BMI group, perception of body weight (PR: 0.57, 95 % CI: 0.49−0.65) was similarly associated with unintentional weight loss and the desire to lose weight (PR: 0.61, 95 % CI: 0.52−0.71). In the overweight/obesity group, the desire to lose weight (PR: 0.48, 95 % CI: 0.42−0.54) showed a stronger association with unintentional weight loss compared to perception of body weight (PR: 0.72, 95 % CI: 0.61−0.84).

## 4 Discussion

Many individuals in the general population have the desire to lose weight and perceive their body weight as too heavy. This trend is evident not only among individuals with a normal weight but is particularly pronounced in those with overweight or obesity, where the vast majority express a desire to lose weight and perceive themselves as too heavy.

Unintentional weight loss, a critical question in the identification of malnutrition risk, appears to be less commonly reported in individuals with the desire to lose weight or those who perceive themselves as too heavy, regardless of BMI status.

Due to the absence of a gold standard in our database, it remains uncertain whether the lower prevalence of unintentional weight loss is an underestimation caused by misclassification or genuinely reflects a lower prevalence among individuals who desire to lose weight or perceive themselves as too heavy. In our analyses, we adjusted for numerous potential confounders, and we still observed a strong association between the desire to lose weight/perception of body weight and unintentional weight loss. Since no plausible mechanism suggests that the desire to lose weight or perception of own body weight could serve as protective factors against experiencing unintentional weight loss, we carefully conclude that the observed lower prevalence is likely due to misclassification.

One of the main reasons for the misclassification could be due to how the unintentional weight loss question was formulated in the Lifelines questionnaire: “*Have you lost a lot of weight recently without wanting to (*6 kg *in* 6 months *or* 3 kg *in one month)?*”. The phrase ‘*without wanting*’ is challenging to interpret negatively for individuals who desire to lose weight or perceive themselves as too heavy. Alternative formulations of unintentional weight loss might yield varied responses within the same population. Given that our data show a significant portion of the population, particularly those with overweight or obesity, express a desire to lose weight, it is essential to examine how different interpretations of unintentional weight loss are perceived and understood. At this moment, malnutrition screening tools use different words to express unintentional weight loss, like ‘unwanted’, ‘unplanned’, and ‘weight loss without trying’. The phrase ‘involuntary weight loss’ is never used in these screening tools. Studies are needed to determine which phrasing is most effective in identifying individuals at risk.

In the Lifelines study, participants responded to the question about unintentional weight loss independently at home, without input from a healthcare professional. In contrast, malnutrition screening tools used in general practice are typically completed in collaboration with a healthcare provider. This difference in context may have influenced the responses, as clearer guidance on interpreting the question could potentially have impacted the results. However, it is unclear whether healthcare professionals provide additional guidance on these questions in daily practice. Our data suggest that healthcare providers should be mindful of potential misclassification. Providing additional clarification about the meaning of ‘*unintentional weight loss*’ is important, as has already been shown in a previous study [5], and may be even more important in individuals with overweight or obesity, where the desire to lose weight is highly prevalent. Perhaps a combination of questions on unintentional weight loss, desire to lose weight and perception of own body weight will be the best approach to identify those with ‘true unintentional weight loss’. Nevertheless, further research is needed to confirm this.

In our study, we used data from the first wave of Lifelines (2007−2013), which is now relatively old. This issue of misclassification may be even more relevant today, as the prevalence of overweight and obesity has increased, along with a potential rise in the desire to lose weight, influenced in part by fat shaming in daily life and on social media. A limitation of the data used, is that it was not specifically designed for our research objectives. For example, a question about percentage weight loss, as recommended by GLIM [7] might have been more relevant, particularly for individuals with higher body weights [3].

## 5 Conclusion

Our findings underscore the challenges of accurately identifying unintentional weight loss in individuals who desire to lose weight or perceive themselves as too heavy. Unintentional weight loss was reported half as often in these participants compared to those who did not desire to lose weight, irrespective of BMI classification. These individuals likely interpret unintentional weight not as unwanted. However, the issue of not reporting unintentional weight loss is more pronounced in individuals with overweight or obesity compared to those with normal weights, as a significantly larger proportion express a desire to lose weight or perceive their body weight as too heavy.

Almost all malnutrition screening tools include a question on unintentional weight loss. Responses to this question are thus likely to be underreported, leading to an underestimation of the risk of malnutrition. A better understanding is needed to determine how unintentional weight loss can be accurately identified in individuals with the desire to lose weight or those who perceive themselves as too heavy.

## Figures and Tables

**Table 1:** Associations between desire to lose weight and unintentional weight loss, stratified by BMI group.

	Normal BMI			Overweight/obesity		
	No unintentional weight loss	Unintentional weight loss	Total	No unintentional weight loss	Unintentional weight loss	Total
Desired to lose weight	16,998 (97.6 %)	422 (2.4 %)	17,420 (30.3 %)	52,880 (97.8 %)	1185 (2.2 %)	54,065 (80.1 %)
Did not desire to lose weight	37,707 (94.0 %) 54,705 (95.1 %) PR: 0.40 (95%CI: 0.36; 0.45) Adjusted PR: 0.40 (95%CI: 0.36; 0.45)^a^	2394 (6.0 %) 2816 (4.9 %)	40,101 (69.7 %) 57,521	12,856 (95.5 %) 65,736 (97.3 %) PR: 0.49 (95%CI: 0.45; 5.40) Adjusted PR: 0.42 (95%CI: 0.38; 0.42)^a^	601 (4.5 %) 1786 (2.6 %)	13,457 (19.9 %) 67,522

a Adjusted for age, gender, arthritis, burnout, COPD, diabetes, cancer, heart attack, employment status, diet score, stress level and rand scores for physical function, health perception, vitality status, mental health, pain, social interactions.

**Table 2:** Associations between perception of body weight and unintentional weight loss, stratified by BMI group.

	Normal BMI			Overweight/obesity		
	No unintentional weight loss	Unintentional weight loss	Total	No unintentional weight loss	Unintentional weight loss	Total
Too heavy	21,087 (97.4 %)	552 (2.6 %)	21,639 (37.9 %)	60,996 (97.6 %)	1517 (2.4%)	62,513 (92.7 %)
Just right/too light	33,134 (93.6 %) 54,221 (95.1 %) PR: 0.40 (95%CI: 0.37; 0.44) Adjusted PR: 0.40 (95%CI: 0.36; 0.45)^a^	2256 (6.4 %) 2808 (4.9 %)	35,390 (62.1 %) 57,029	4680 (94.6 %) 65,676 (97.4 %) PR: 0.45 (95%CI: 0.40; 0.51) Adjusted PR: 0.43 (95%CI: 0.37; 0.49)^a^	266 (5.4 %) 1783 (2.6 %)	4946 (7.3 %) 67,459

a Adjusted for age, gender, arthritis, burnout, COPD, diabetes, cancer, heart attack, employment status, diet score, stress level and rand scores for physical function, health perception, vitality status, mental health, pain, social interactions.

## References

[1] Leij-Halfwerk S, Verwijs MH, van Houdt S, Borkent JW, Guaitoli P, Pelgrim T (2019). Prevalence of protein-energy malnutrition risk in European older adults in community, residential and hospital settings, according to 22 malnutrition screening tools validated for use in adults ≥65 years: a systematic review and meta-analysis. Maturitas.

[2] Stival C, Lugo A, Odone A, Van den Brandt PA, Fernandez E, Tigova O (2022). Prevalence and correlates of overweight and obesity in 12 European Countries in 2017–2018. Obes Facts.

[3] Mwala NN, Borkent JW, van der Meij BS, de van der Schueren MA (2024). Challenges in identifying malnutrition in obesity; an overview of the state of the art and directions for future research. Nutr Res Rev.

[4] de van der Schueren MA, Jager-Wittenaar H (2022). Malnutrition risk screening: new insights in a new era. Clin Nutr.

[5] Visser M, Sealy M, Leistra E, Naumann E, De van der Schueren M, Jager-Wittenaar H (2024). The malnutrition awareness scale for community-dwelling older adults: development and psychometric properties. Clin Nutr.

[6] Sijtsma A, Rienks J, van der Harst P, Navis G, Rosmalen JG, Dotinga A (2022). Cohort profile update: Lifelines, a three-generation cohort study and biobank. Int J Epidemiol.

[7] Cederholm T, Jensen G, Correia M, Gonzalez MC, Fukushima R, Higashiguchi T (2019). GLIM criteria for the diagnosis of Malnutrition–A consensus report from the global clinical nutrition community. J cachexia, sarcopenia muscle.

[8] Knol MJ, Le Cessie S, Algra A, Vandenbroucke JP, Groenwold RH (2012). Over-estimation of risk ratios by odds ratios in trials and cohort studies: alternatives to logistic regression. CMAJ (Can Med Assoc J).

[9] Vinke PC, Corpeleijn E, Dekker LH, Jacobs DR, Navis G, Kromhout D (2018). Development of the food-based lifelines diet score (LLDS) and its application in 129,369 lifelines participants. Eur J Clin Nutr.

[10] Hays RD, Sherbourne CD, Mazel RM (1993). The rand 36-item health survey 1.0. Health Econ.

